# The clinical application of a scoring protocol to select endarterectomy or stenting for carotid artery stenosis

**DOI:** 10.1038/s41598-022-08807-8

**Published:** 2022-03-18

**Authors:** Joonho Chung, Jung-Jae Kim, Yong Bae Kim, Sang Hyun Suh, Kyung-Yul Lee

**Affiliations:** 1 Department of Neurosurgery, Severance Hospital, Yonsei University College of Medicine, Seoul, Republic of Korea; 2 Department of Neurosurgery, Ewha Womans University Seoul Hospital, Seoul, Republic of Korea; 3 Department of Radiology, Gangnam Severance Hospital, Yonsei University College of Medicine, Seoul, Republic of Korea; 4 Department of Neurology, Gangnam Severance Hospital, Severance Institute for Vascular and Metabolic Research, Yonsei University College of Medicine, 211, Eonjuro, Gangnam-gu, Seoul, 135-720 Republic of Korea

**Keywords:** Neuroscience, Neurology

## Abstract

Previously we described the protocol-based decision for choosing the proper surgical treatment option for carotid stenosis. The objective of this study is to describe our experiences of using this scoring protocol in the selection of endarterectomy or stenting for carotid stenosis. Between October 2014 and March 2018, the scoring protocol was applied to a total of 105 consecutive patients. Eighty (76.2%) patients had symptomatic stenosis ≥ 50%, and 25 (23.8%) patients had asymptomatic stenosis ≥ 80%. We also speculated about how effectively the protocol worked in the real clinical setting. Stenting was performed in 73 patients and endarterectomy in 32 patients. Overall, 98 (93.3%) patients were treated according to the protocol, while the protocol was violated in seven (6.7%) patients. Sixty-one (58.1%) patients received treatments that were decided by the protocol. There were 37 (35.2%) patients who had the same score for both treatment options. Among these patients, 28 patients underwent stenting and nine patients underwent endarterectomy. In the stenting cases, 90.4% of the patients followed the protocol and violations occurred in 9.6%. In the endarterectomy cases, all of the patients followed the protocol. Overall, one patient had a procedure-related complication without morbidity. During the 12-month follow-ups, there were no restenoses or major strokes. Minor strokes were diagnosed in three (2.8%) patients. In patients with carotid artery stenosis, stenting and endarterectomy should be considered simultaneously together, not against each other. Our scoring protocol can be used to weigh these options and applied in clinical practice.

## Introduction

Carotid artery stenosis accounts for 7–20% of all ischemic strokes^1–4^. Currently, there are two surgical treatment options for carotid artery stenosis. These include carotid artery stenting (CAS) and carotid endarterectomy (CEA). Large trials have demonstrated that each surgical procedure has its own benefits and risks^4–11^. Based on the American guidelines from 2014, CEA is recommended as the first choice of treatment for patients with severe carotid stenosis^12^. However, there are some revised and new recommendations that suggest CAS can be an alternative to CEA if the periprocedural stroke or death rate is < 6%. This recommendation describes the possibility of performing carotid stenting as the first choice of treatment. In addition, CAS is equivalent to CEA in younger patients; therefore, it may be the first treatment option in these cases as well^12^.

We previously described a protocol-based decision for choosing the proper surgical treatment of carotid artery stenosis^13^. Our group developed a scoring protocol that included absolute indications and favorable indications for each treatment option (CEA or CAS) (Table 1). Each absolute indication is scored with three points, while each favorable indication offers one point. Based on the highest scores, the proper treatment option can be selected. If the score was the same for CEA and CAS, the treatment option is at the discretion of the patient. Since this paper was published in 2015, we have applied the protocol to our patients. Therefore, the purpose of this study was to describe our experience of using this scoring protocol to select CEA or CAS for carotid artery stenosis, and to evaluate the associated clinical outcomes.

**Table 1:** Protocol for selection of a proper treatment option for carotid stenosis.

Absolute CAS	Favorable CAS	Absolute CEA	Favorable CEA
Heart failure (EF ≤ 30% on TEE)	Stable angina including a history of coronary stenting with 30% < EF ≤ 40%	Failure of DSA	Renal failure without hemodialysis
Myocardial infarction within 4 weeks	Poor collateral flow of anterior communicating artery	Severe vascular disease of endovascular access	Complicated atheroma on the ascending aortic arch
Need for open heart surgery within 6 weeks	Carotid artery tandem lesions	Allergic reaction to contrast	Type III aortic arch
Pulmonary dysfunction (PFT, FEV1 or DLCO ≤ 50%)	Emergency	Heavy calcification: concentric circumferential ≥ 270°	String sign
Contralateral carotid artery occlusion			Ulcerated lesion
Contralateral laryngeal paralysis			The length of the lesion ≥ 30 mm
High stenosis above C2 or low stenosis below clavicle			Thrombus-containing stenosis on DSA
Previous radiation of the neck			Moderate calcification (90° ≤ circumference < 270°) with calcification thickness ≥ 3 mm
Previous radical neck surgery			
Restenosis after CEA			
Former tracheostomy			

*CAS* carotid artery stenting, *CEA* carotid endarterectomy, *DLCO* diffusion capacity of the lung for carbon monoxide, *DSA* digital subtraction cerebral angiography, *EF* ejection fraction, *FEV1* forced expiratory volume in 1 s, *PFT* pulmonary function test, *TEE* trans esophageal echocardiogram.

## Results

Among the 105 patients, the mean age was 71.6 years (age range: 41 to 88 years) and 84 (80.0%) patients were male (Table 2). The mean degree of carotid stenosis at the lesion was 83.8%. CAS was performed in 73 patients and CEA in 32 patients. There were no significant differences in age, sex ratio, hypertension, diabetes, smoking, dyslipidemia, presenting symptoms, or degree of stenosis between the CAS and CEA cases.

**Table 2:** Demographic characteristics and clinical results up to 12-month follow-up.

	Total (n = 105)	Stenting (n = 73)	Endarterectomy (n = 32)	P-value
**Age, year**				
Mean ± SD	71.6 ± 8.2	71.0 ± 8.9	73.0 ± 6.2	0.348*
Distribution				0.572
< 70	38 (36.2)	29 (39.7)	9 (28.1)	
≥ 70	67 (63.8)	44 (60.3)	23 (71.9)	
**Sex**				0.104
Male	84 (80.0)	64 (87.7)	20 (62.5)	
Female	21 (20.0)	9 (12.3)	12 (37.5)	
Hypertension	80 (76.2)	56 (76.6)	24 (75.0)	0.716
Diabetes	43 (41.0)	30 (41.1)	13 (40.6)	0.841
Smoking	32 (30.5)	27 (37.0)	5 (15.6)	0.272
Dyslipidemia	58 (55.2)	40 (54.8)	18 (56.3)	0.659
**Presentation**				0.726
Previous stroke	3 (2.9)	2 (2.7)	1 (3.1)	
Hemispheric symptoms	69 (65.7)	49 (67.1)	20 (62.5)	
Retinal symptoms	8 (7.6)	5 (6.8)	3 (9.4)	
Asymptomatic	25 (23.8)	17 (23.2)	8 (25.0)	
**Degree of stenosis**				
Mean ± SD	83.8 ± 6.9	83.5 ± 6.6	84.5 ± 7.9	0.865*
Distribution				0.658
70–79%	15 (14.3)	9 (12.3)	6 (18.8)	
80–89%	33 (31.4)	23 (31.5)	10 (31.3)	
90–99%	48 (45.7)	34 (46.6)	14 (43.8)	
Technical success	100%	100%	100%	NA
Procedure-related complication	1 (0.9)	1 (1.4)	0 (0)	0.329
Minor stroke	3 (2.8)	2 (2.7)	1 (3.1)	0.618
Major stroke	0 (0)	0 (0)	0 (0)	NA
Mortality rate	0 (0)	0 (0)	0 (0)	NA
Restenosis	0 (0)	0 (0)	0 (0)	NA

*MI* myocardial infarction, *NA* not applicable, *SD* standard deviation.

P-value, Chi-square test or Fisher exact test.

*Student t-test.

**Figure 1:**
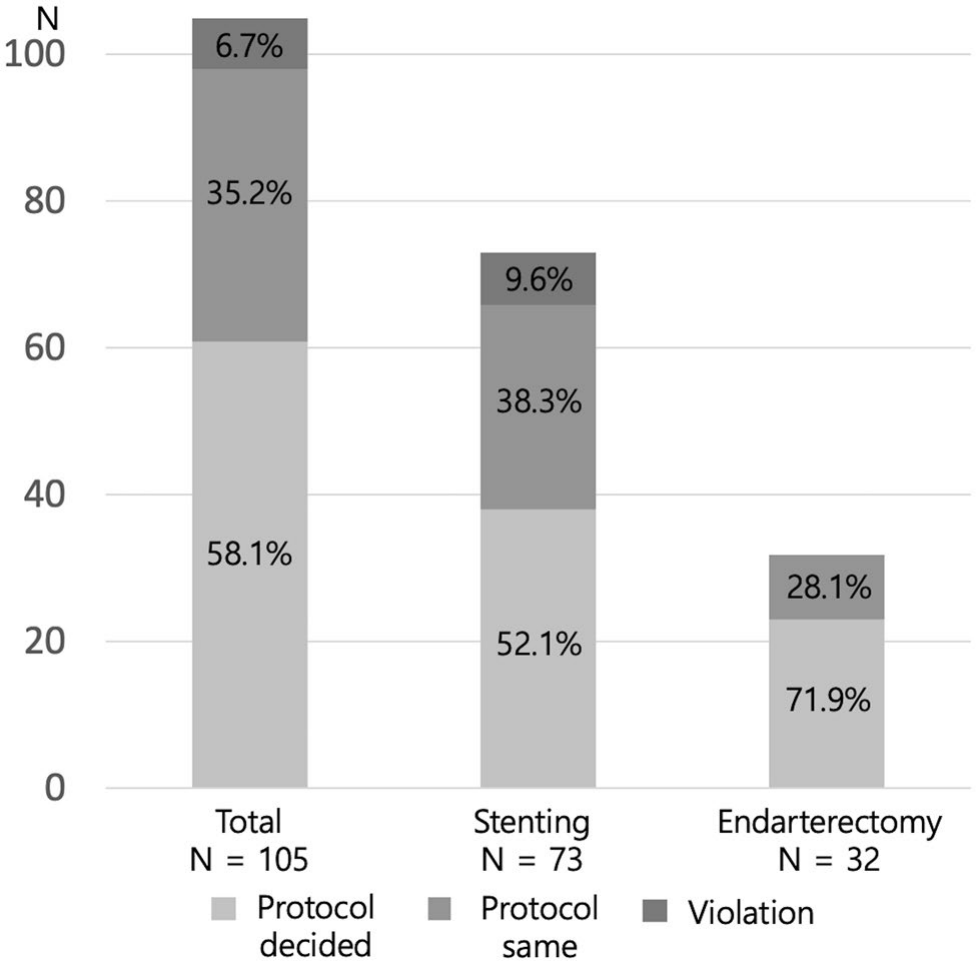
The proportion of treatments applied. A total of 98 (93.3%) patients were treated following protocol. Among them, 61 (58.1%) patients received treatments decided by the protocol. There were violations in seven (6.7%) patients. Although these patients had higher scores for endarterectomy, they all received carotid stenting. There were 37 (35.2%) patients who had equal scores for endarterectomy and carotid stenting also tended to choose stenting over endarterectomy.

Ninety-eight (93.3%) patients were treated following the protocol. The protocol was violated in seven (6.7%) patients who received CAS, although they had higher scores for CEA. The protocol decided the surgical procedure that was performed in 61 (58.1%) patients. However, 37 (35.2%) patients had the same score for both treatment options (Fig. 1). Among them, 28 patients underwent CAS and nine patients underwent CEA.

**Figure 2:**
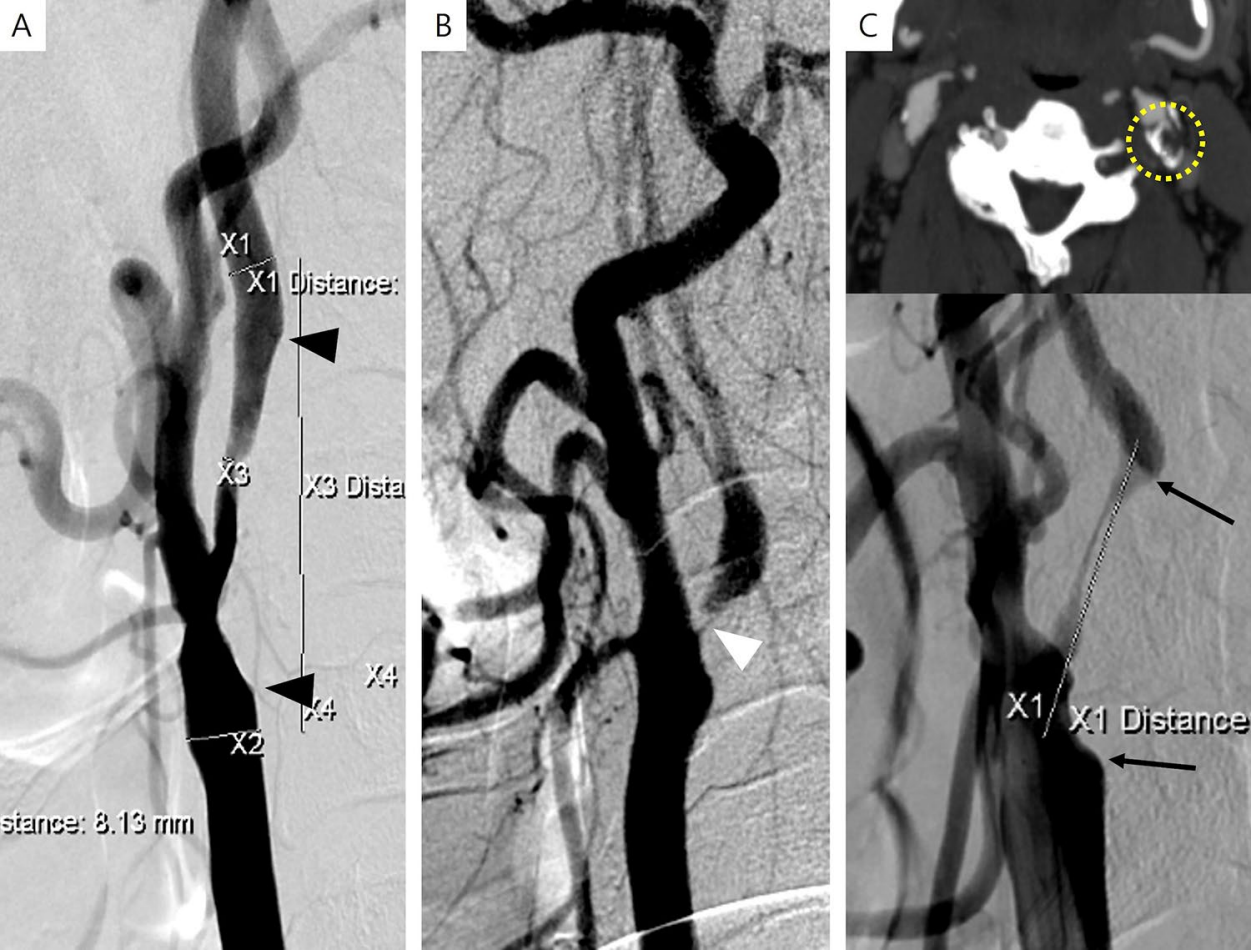
Violation cases. Patients with higher scores for endarterectomy chose to receive stenting rather than endarterectomy. (**A**) A 65-year old male with right symptomatic carotid artery stenosis (79% stenosis) acquired 1 point for favorable CEA [CAS(0) CEA(1)] due to the length of the lesion ≥ 30 mm (*indicated by black arrowheads*). (**B**) A 51-year old female with left symptomatic carotid artery stenosis (99% stenosis) acquired 1 point for favorable CEA [CAS(0) CEA(1)] due to the string sign (*indicated by a white arrowhead*). (**C**) A 59-year old male with left symptomatic stenosis (90% stenosis) acquired 2 points for favorable CEA [CAS(0) CEA(2)] due to the length of the lesion ≥ 30 mm (*indicated by black arrows*) and moderate calcification (90° ≤ circumference < 270°, *indicated by a yellow dot-circle*).

In the stenting cases, 66 (90.4%) patients followed the protocol, while it was violated in seven patients who should have undergone CAS (Fig. 2). Among 38 (52.1%) patients in whom stenting was decided by the protocol, nine patients had scores with absolute indications and 29 patients had scores with favorable indications for CAS. Among the 28 (38.3%) patients in whom the scores were equally for both treatment options and chose to undergo stenting, seven had one point for favorable CAS and 21 patients did not have any such points. Among the seven violation cases, one patient had one point for favorable CAS, but had five points from one absolute CEA and two favorable CEA. Another patient had one point for favorable CAS but had two points for two favorable CEA. The other five patients did not have any points for CAS. Two patients had two points for two favorable CEA, and three patients had one point for one favorable CEA (Table 3).

**Table 3:** Number of cases according to points from the protocol.

	Case (n)	Points	Absolute CAS (3 points)	Favorable CAS (1 point)	Absolute CEA (3 point)	Favorable CEA (1 points)
**CAS cases (n = 73)**						
Protocol decided (n = 38)						
Absolute CAS (n = 9)	1	CAS(6) CEA(0)	2	0	0	0
	2	CAS(4) CEA(0)	1	1	0	0
	6	CAS(3) CEA(0)	1	0	0	0
Favorable CAS (n = 29)	1	CAS(3) CEA(0)	0	3	0	0
	1	CAS(2) CEA(1)	0	2	0	1
	27	CAS(1) CEA(0)	0	1	0	0
Protocol same (n = 28)	7	CAS(1) CEA(1)	0	1	0	1
	21	CAS(0) CEA(0)	0	0	0	0
Violation (n = 7)	1	CAS(1) CEA(5)	0	1	1	2
	1	CAS(1) CEA(2)	0	1	0	2
	2	CAS(0) CEA(2)	0	0	0	2
	3	CAS(0) CEA(1)	0	0	0	1
**CEA cases (n = 32)**						
Protocol decided (n = 23)						
Absolute CEA (n = 11)	1	CAS(0) CEA(5)	0	0	2	1
	3	CAS(0) CEA(4)	0	0	1	1
	1	CAS(3) CEA(4)	1	0	1	1
	6	CAS(0) CEA(3)	0	0	1	0
Favorable CEA (n = 12)	1	CAS(0) CEA(2)	0	0	0	2
	11	CAS(0) CEA(1)	0	0	0	1
Protocol same (n = 9)	2	CAS(3) CEA(3)	1	0	1	0
	1	CAS(1) CEA(1)	0	1	1	1
	6	CAS(0) CEA(0)	0	0	0	0

*CAS* carotid artery stenting, *CEA* carotid endarterectomy.

In the endarterectomy cases, all of the patients followed the protocol. There were no violations. Among the 23 (71.9%) patients in whom endarterectomy was decided by the protocol, 11 patients had scores for absolute CEA and 12 patients had scores for favorable CEA. Among the nine (28.1%) patients who had the same score on both treatment options and chose endarterectomy, two patients had three points for one absolute CEA, one patient had one point for one favorable CEA. Six patients did not have any points (Table 3).

The rate of technical success was 100%. There was one procedure-related complication, which was a puncture site hematoma (in a stenting case) that required extended hospital stay. On the 12-month follow-ups, there were no restenoses or major strokes. Minor stroke occurred in three (2.8%) patients within 30 days. Two (2.7%) of the minor strokes occurred in stenting cases, and one (3.1%) in an endarterectomy case (p = 0.618).

## Discussion

In this study, we verified the real-world application of our scoring protocol to patients with carotid stenosis. In our patient population, a majority (93.3%) of the treatment options were chosen by the protocol. Among those in whom the scores were equal and those who should have undergo CEA according to the protocol, patients tended to choose CAS over CEA. Seven patients who would have benefitted from CEA (based on the protocol) actually underwent CAS. We suspect that it is unnecessary to determine which treatment arm is better than the other between CAS and CEA. We believe that no future efforts should be made to choose one or the other. Instead, we should identify which treatment option is more appropriately tailored to each patient. The consideration of both CEA and CAS simultaneously (with respect to each patient) is expected to improve clinical outcomes overall.

Our protocol was developed using clinically relevant preoperative factors (with regard to risk/benefit) that had been identified in several previous articles^13^. Among the 192 articles that we found on PubMed and Medline, 28 were selected as references and they all met the following criteria: (1) single or multiple randomized clinical trials; (2) review articles in journals with high impact factors (≥ 6); or (3) well-designed case–control studies including a large number of patients. We made a general outline for our protocol based on these studies. We then specified the exact indicative values for each factor suitable for our institution. For example, we gave one point for moderate calcification around the carotid stenosis with a concentric circumference of 90–270 degrees with maximal thickness of calcified plaque ≥ 3 mm as favorable for CEA. We also gave one point for lesion length ≥ 30 mm as favorable for CEA^14–20^. However, those factors are not contraindications to perform CAS. Although we gave these points in favor of CEA, we expected patients to prefer CAS by choice. This method allowed us to assess the real-world application of our protocol.

Among the seven violation cases, all patients declined to undergo CEA. These patients preferred CAS because it is minimally invasive, produces less of a scar, and does not require general anesthesia. It is certainly possible that emotions and indirect outside influences affected patients’ decision to undergo CAS over what was recommended by the medical evidence. These same factors may have also influenced patients’ decisions when the scores were equal between CEA and CAS. Among the 37 patients with equal scores on the protocol, 28 (75.7%) underwent CAS. We preferred CAS to CEA in emergent situations, such as acute ischemic stroke. We performed CAS in 11 patients during intra-arterial thrombectomy or diagnostic angiography in emergent situations.

Based on previous large trials^4–11^, the American guidelines recommended CEA as the initial treatment option in patients with symptomatic severe stenosis in 2014^12^. However, CAS has become an option based on several new recommendations if the periprocedural stroke or death rate is < 6%. This recommendation allows patients to be treated with CAS if there is no available vascular surgeon to perform CEA. In addition, CAS is equivalent to CEA in young patients. The clinical outcomes from our series cannot be directly compared with those of previous studies due to the purpose and the design of this study. However, our study does provide evidence that protocol-based decision making is safe and produces comparable outcomes to those from previous trials and recommendations^4–12^. Although our sample size was very small, there was no permanent neurological deficits or deaths in our series. This suggests that our scoring protocol could be applicable to the real-world clinical setting. The 30-day mortality (0%), major stroke (0%), minor stroke (3.1%) and myocardial infarction (0%) rates were similar or better in our series than those from previous large trials. Our clinical outcomes were also comparable between both treatment options. The 12-month outcomes of the 105 consecutive patients in this study might be sufficient to recommend our protocol for determining the appropriate treatment of carotid stenosis.

We have considered additional factors for modification of our protocol. First, atrial fibrillation with anticoagulation may belong to the category of “favorable CEA,” because dual antiplatelet therapy for CAS may increase the bleeding risk. In addition, acute ischemic stroke requiring mechanical thrombectomy combined with large artery occlusions due to severe carotid stenosis may belong to the category of “favorable CAS,” given its emergent nature. Furthermore, the string sign requires more precise indicative values. We may give one point to CEA if the length of the string sign is ≥ 2 cm. Otherwise, both treatment options are applicable. We did not experience restenosis after CAS in this present study. However, if we had, it would likely belong the category of “favorable CAS.” Finally, we should have included an indication to choose CEA or CAS if absolute CAS and absolute CEA are pitted against each other. We recognize that our small sample size is a limitation of this study. Still, we suspect that it is sufficient to show the tendency of real-world practice with application of our protocol and the shift toward CAS and minimally invasive treatment.

In the treatment of patients with carotid artery stenosis, CAS and CEA should be considered simultaneously together, not against each other. In the present study, a majority of the treatment options were chosen by the protocol. Among those in whom the scores were equal and those who should have undergo CEA according to the protocol, patients tended to choose CAS over CEA. Our scoring protocol can be used to weigh these options and applied in clinical practice.

## Methods

This retrospective study was approved and the requirement for informed consent was waived by Yonsei University Health System, Severance Hospital, Institutional Review Board (IRB number, 2021-2179-002). All methods were performed in accordance with the relevant guidelines and regulations. Between October 2014 and March 2018, the scoring protocol was applied to 105 consecutive patients. Eighty (76.2%) patients had symptomatic stenosis ≥ 50%, and 25 (23.8%) patients had asymptomatic stenosis ≥ 80% measured by the North American Symptomatic Carotid Endarterectomy Trial (NASCET) criteria.

We established the scoring protocol based on the following factors; difficulties of anatomic approach to the carotid artery either by CEA or CAS; cardiopulmonary function (evaluated by echocardiogram and pulmonary function testing); existence of renal failure; previous history of neck surgery or radiation; contralateral laryngeal paralysis; allergic reaction to contrast medium; vascular access for diagnostic digital subtraction cerebral angiography (DSA); calcification around the carotid artery stenosis (evaluated by carotid artery computed tomography); complicated atheroma on the ascending aortic arch (evaluated by echocardiogram); string sign; ulcerated stenosis (evaluated by DSA when it was seen as a crater from the lumen into a stenotic plaque); the length of the lesion; and the existence of tandem stenosis (multifocal stenosis from the proximal cervical to the distal internal carotid artery or to the ipsilateral middle cerebral artery); contralateral carotid artery occlusion; and poor collateral flow of the anterior communicating artery.

As shown in Table 1, each treatment option has absolute and favorable indications. A simple numerical score was assigned for each indication. The absolute indications were weighted three times higher than were the favorable indications. We chose this scoring system because the absolute indications have been debated in several articles, including major randomized controlled trials, and are considered as "absolute." Given the prior research, we decided that one absolute indication should be considered more important than two favorable indications.

We evaluated how the protocol worked in the real clinical application and evaluated the clinical patient outcomes. The clinical outcomes were assessed with regard to procedure-related complications, minor stroke, major stroke (morbidity), and mortality within 12 months. Any new neurologic deficits were scored using the National Institutes of Health Stroke Scale (NIHSS). A minor stroke was defined by a new neurological event lasting ≥ 24 h but resolving within 30 days, and with an increase in the NIHSS less than 3. A major stroke was defined as a new neurological event that lasted ≥ 24 h, with an increase in the NIHSS greater than 3. The patients were clinically followed-up for a mean of 33.8 months (range: 12–53 months).

### Surgical management

All of the CEA procedures were performed under general anesthesia with somatosensory evoked potential and/or electroencephalography monitoring. If the preoperative DSA showed that there was insufficient cross-filling during the Matas Test and the Alcock Test, then carotid shunts were used during the CEA. A local lidocaine infiltration at the carotid sinus was performed when the patient’s heart rate was < 40 bpm. A local injection was performed in 15 (46.9%) of 32 patients in this study. After plaque removal, we preferred continuous-suture closure for artery repair rather than carotid patch angioplasty.

Carotid artery stenting was routinely performed after administration of a local anesthetic and with strict blood pressure monitoring. A distal embolic protection device and a self-expanding nitinol stent were used in all 73 patients undergoing CAS.

### Statistical analysis

All statistical analyses were performed using IBM SPSS Statistics version 22.0 (IBM, Armonk, New York, USA) in consultation with a biostatistician. Student t-test was used for numeric variables. Chi-square test or Fisher exact test were used for nominal variables. A *P* value < 0.05 was considered statistically significant.

## Data Availability

All data generated during and/or analyzed during the current study are available from the corresponding author on reasonable request.

## References

[1] Flaherty ML (2013). Carotid artery stenosis as a cause of stroke. Neuroepidemiology.

[2] Dharmakidari S, Bhattacharya P, Chaturvedi S (2017). Carotid artery stenosis: Medical therapy, surgery, and stenting. Curr. Neurol. Neurosci. Rep..

[3] Dyken, M. Stroke risk factors in prevention of stroke. In (eds Norris, J. W. & Hachinski, V. C.) 83–102 (Springer, 1991)

[4] CAVATAS Investigators (2001). Endovascular versus surgical treatment in patients with carotid stenosis in the Carotid and Vertebral Artery Transluminal Angioplasty Study (CAVATAS): A randomized trial. Lancet.

[5] North American Symptomatic Carotid Endarterectomy Trial Collaborators (1991). Beneficial effect of carotid endarterectomy in symptomatic patients with high-grade stenosis. N. Engl. J. Med..

[6] European Carotid Surgery Trialists’ Collaborative Group (1998). Randomised trial of endarterectomy for recently symptomatic carotid stenosis: Final results of the MRC European Carotid Surgery Trial (ECST). Lancet.

[7] Yadav JS (2004). Protected carotid-artery stenting versus endarterectomy in high-risk patients. N. Engl. J. Med..

[8] SPACE Collaborative Group (2006). 30 day results from the SPACE trial of stent-protected angioplasty versus carotid endarterectomy in symptomatic patients: A randomized non-inferiority trial. Lancet.

[9] Mas JL (2006). Endarterectomy versus stenting in patients with symptomatic severe carotid stenosis. N. Engl. J. Med..

[10] International Carotid Stenting Study investigators (2010). Carotid artery stenting compared with endarterectomy in patients with symptomatic carotid stenosis (International Carotid Stenting Study): An interim analysis of a randomized controlled trial. Lancet.

[11] Brott TG (2010). Stenting versus endarterectomy for treatment of carotid-artery stenosis. N. Engl. J. Med..

[12] Kernan WN (2014). Guidelines for the prevention of stroke in patients with stroke and transient ischemic attack: A guideline from healthcare professionals from the American Heart Association/American Stroke Association. Stroke.

[13] Jang EW, Chung J, Seo KD, Suh SH, Kim YB, Lee KY (2015). A protocol-based decision for choosing a proper surgical treatment option for carotid artery stenosis. J. Cerebrovasc. Endovasc. Neurosurg..

[14] Gray WA (2007). The CAPTURE registry: Predictors of outcomes in carotid artery stenting with embolic protection for high surgical risk patients in the early post-approval setting. Catheter Cardiovasc. Interv..

[15] Groschel K, Schnaudigel S, Ernemann U, Wasser K, Kastrup A (2008). Size matters! Stent-length is associated with thrombembolic complications after carotid artery stenting. Stroke.

[16] Hofmann R, Niessner A, Kypta A, Wasser K, Kastrup A (2006). Risk score for peri-interventional complications of carotid artery stenting. Stroke.

[17] Roubin GS, Iyer S, Halkin A, Vitek J, Brennan C (2006). Realizing the potential of carotid artery stenting: Proposed paradigms for patient selection and procedural technique. Circulation.

[18] Setacci C, Chisci E, Setacci F, Iacoponi F, de Donato G, Rossi A (2010). Siena carotid artery stenting score: A risk modelling study for individual patients. Stroke.

[19] Shankar JJ, Zhang J, dos Santos M, Lesiuk H, Mohan R, Lum C (2012). Factors affecting long-term restenosis after carotid stenting for carotid atherosclerotic disease. Neuroradiology.

[20] Wimmer NJ, Yeh RW, Cutlip DE, Mauri L (2012). Risk prediction for adverse events after carotid artery stenting in higher surgical risk patients. Stroke.

